# Revisiting Diagnosis and Treatment of Hypertrophic Cardiomyopathy: Current Practice and Novel Perspectives

**DOI:** 10.3390/jcm12175710

**Published:** 2023-09-01

**Authors:** Andrea Ottaviani, Davide Mansour, Lorenzo V. Molinari, Kristian Galanti, Cesare Mantini, Mohammed Y. Khanji, Anwar A. Chahal, Marco Zimarino, Giulia Renda, Luigi Sciarra, Francesco Pelliccia, Sabina Gallina, Fabrizio Ricci

**Affiliations:** 1 Department of Neuroscience, Imaging and Clinical Sciences, “G. D’Annunzio” University of Chieti-Pescara, 66100 Chieti, Italy; 2 Barts Heart Centre, Barts Health NHS Trust, London EC1A 7BE, UK; 3 Newham University Hospital, Barts Health NHS Trust, London E13 8SL, UK; 4 NIHR Barts Biomedical Research Centre, William Harvey Research Institute, Queen Mary University of London, London EC1A 7BE, UK; 5 Inherited Cardiovascular Diseases, WellSpan Health, Lancaster, PA 17605, USA; 6 Cardiac Electrophysiology, Cardiovascular Division, Hospital of the University of Pennsylvania, Philadelphia, PA 17605, USA; 7 Heart Department, SS. Annunziata Hospital, ASL 2 Abruzzo, 66100 Chieti, Italy; 8 Department of Life, Health and Environmental Sciences, University of L’Aquila, 67100 L’Aquila, Italy; 9 Department of Cardiovascular Sciences, Sapienza University, 00166 Rome, Italy; 10 Department of Clinical Sciences, Lund University, 21428 Malmö, Sweden

**Keywords:** hypertrophic cardiomyopathy, treatment, myosin modulators, genetics, cardiovascular imaging

## Abstract

Sarcomeric hypertrophic cardiomyopathy (HCM) is a prevalent genetic disorder characterised by left ventricular hypertrophy, myocardial disarray, and an increased risk of heart failure and sudden cardiac death. Despite advances in understanding its pathophysiology, treatment options for HCM remain limited. This narrative review aims to provide a comprehensive overview of current clinical practice and explore emerging therapeutic strategies for sarcomeric HCM, with a focus on cardiac myosin inhibitors. We first discuss the conventional management of HCM, including lifestyle modifications, pharmacological therapies, and invasive interventions, emphasizing their limitations and challenges. Next, we highlight recent advances in molecular genetics and their potential applications in refining HCM diagnosis, risk stratification, and treatment. We delve into emerging therapies, such as gene editing, RNA-based therapies, targeted small molecules, and cardiac myosin modulators like mavacamten and aficamten, which hold promise in modulating the underlying molecular mechanisms of HCM. Mavacamten and aficamten, selective modulators of cardiac myosin, have demonstrated encouraging results in clinical trials by reducing left ventricular outflow tract obstruction and improving symptoms in patients with obstructive HCM. We discuss their mechanisms of action, clinical trial outcomes, and potential implications for the future of HCM management. Furthermore, we examine the role of precision medicine in HCM management, exploring how individualised treatment strategies, including exercise prescription as part of the management plan, may optimise patient outcomes. Finally, we underscore the importance of multidisciplinary care and patient-centred approaches to address the complex needs of HCM patients. This review also aims to encourage further research and collaboration in the field of HCM, promoting the development of novel and more effective therapeutic strategies, such as cardiac myosin modulators, to hopefully improve the quality of life and outcome of patients with sarcomeric HCM.

## 1. Introduction

Hypertrophic cardiomyopathy (HCM) is a hereditary cardiac condition featuring left ventricular hypertrophy (LVH), which abnormal loading conditions cannot fully explain. It has been traditionally viewed as an uncommon condition with few effective treatment alternatives for controlling the possibility of dangerous disease advancement and malignant ventricular arrhythmias [1]. HCM is a common inheritable cardiac disorder usually following an autosomal dominant inheritance pattern. LVH in the absence of cardiovascular diseases occurs in approximately 1:500 subjects in the general population; when both clinical and genetic diagnoses are considered [2], this prevalence increases to 1 case per 200 [3,4,5]. HCM stems from multiple mutations that affect at least 14 genes [6] responsible for sarcomeric proteins. Over 1400 distinct variants in at least 11 genes are known to cause HCM; still, nearly 70% occur either in the MYH7 gene, which encodes for the β-myosin heavy chain, or in the MYBPC3 gene, which encodes for the myosin binding protein C. HCM can also occur as part of a syndrome and might exhibit non-sarcomeric genocopies. It is essential that these are identified, as targeted therapies are available for them, whereas treatments for sarcomeric HCM would be inappropriate. Key distinctions include amyloidosis, Fabry disease, glycogen storage diseases, and syndromic disorders, such as RASopathies, Timothy syndrome, and Emery–Dreifuss muscular dystrophy (FHL1).

### 1.1. Pathophysiology

HCM is characterised by the absence of any abnormal loading conditions while exhibiting LVH. The clinical manifestation of HCM is varied and includes several pathological features, which stem from both the direct functional impacts of the disease-causing mutation and the secondary changes in function that the affected myocardium undergoes in response to it.

HCM features myocardial fibrosis (ranging from microscopic fibrosis within the myocardium to significant macroscopic scarring), abnormalities in small coronary vessels (microvascular remodelling and dysfunction), haphazard organisation of tissue (myocardial disarray), papillary muscle abnormalities (hypertrophy, feathering, apical insertion), and anomalies in the mitral valve (elongated anterior leaflet), as well as ventricular and supraventricular tachyarrhythmias.

The varied genetic makeup of HCM plays a significant role in explaining the wide range of differences seen among patients with HCM. In a study by Beltrami et al., MYBPC3-related HCM showed an increased long-term prevalence of systolic dysfunction compared with MYH7, despite similar outcomes [7]. Such observations suggest different pathophysiology of clinical progression in the two subsets and may prove relevant for understanding of genotype-phenotype correlations in HCM. Additionally, relatives with the same genetic mutation may present with different clinical features. As a result, the management of HCM patients poses a considerable challenge, necessitating tailored and individualised approaches [1].

### 1.2. Obstructive vs. Nonobstructive HCM

Obstructive HCM (oHCM) features LVOT obstruction (LVOTO), defined by the presence of a peak LVOT gradient of ≥30 mmHg by continuous wave Doppler echocardiography, with resting or provoked gradients ≥50 mmHg generally considered to be the threshold for septal reduction therapy in those patients with drug-refractory symptoms [3]. LVOTO is a common finding in HCM patients and may be present during rest or provoked by a Valsalva manoeuvre, exercise, or drug. It is a dynamic consequence, dependent on preload conditions and anatomy, in which the LVOT becomes obstructed and typically affects around 50% of individuals diagnosed with HCM at some stage. In addition, individuals with oHCM often experience symptoms of heart failure (HF), such as shortness of breath, difficulty exercising, and angina. They may also experience syncope or pre-syncope, particularly on exertion or lifting. Since its initial discovery, clinical and preclinical research findings have transformed the understanding of HCM from a rare and dangerous condition to a relatively frequent disease with generally stable progression. Compared to other inherited heart muscle disorders, HCM has lower morbidity and mortality (including sudden death) [1]. While some individuals with HCM may have a typical lifespan, LVOTO, atrial fibrillation, or ventricular arrhythmias can considerably impact each person’s prognosis and quality of life.

### 1.3. HCM Phenocopies

HCM is a genetic cause of cardiomyopathy that is widespread worldwide. Genetic testing advancements have improved the detection of sarcomeric mutations responsible for HCM [8]. However, this has also highlighted the importance of identifying inborn errors of metabolism or metabolic storage disorders that can mimic HCM (phenocopies). Distinguishing these conditions can be challenging. HCM phenocopies are common, and it is crucial to differentiate them early due to significant differences in prognosis, management, and natural history compared to sarcomeric HCM (Table 1).

- **Anderson–Fabry disease:** this is a genetic disorder caused by mutations in the α-galactosidase A (GLA) gene that results in the accumulation of glycosphingolipids in various systems of the body. The disease is known to cause LVH, which can be diagnosed through echocardiography in almost 50% of male patients aged 30–40 [9]. LVH in Anderson–Fabry Disease happens due to an abnormal build-up of glycosphingolipids, leading to reduced α-galactosidase A activity. Most patients also exhibit abnormal ECG results, with voltage criteria for LVH, short PR interval and conduction disorders. Echocardiography shows concentric LVH, diastolic dysfunction, and systolic dysfunction in the later stages of the disease. Cardiovascular magnetic resonance (CMR) scans also show concentric LVH, with a late gadolinium enhancement (LGE) of the basal inferolateral wall being a characteristic feature. Moreover, LGE in these patients increases the risk of major cardiovascular events [10,11]. Most Fabry patients exhibit concentric LVH [12]; however, asymmetrical septal and apical hypertrophy can be observed. Cardiovascular involvement can be highly variable and mutation dependent, also affecting the eligibility of precision therapies such as oral chaperone therapy. Enzyme replacement therapy is dependent on serum alpha-1-galactosidase levels.
- **Cardiac amyloidosis:** this is a cardiomyopathy caused by the deposition of amyloid protein outside of the heart cells, affecting the myocardium. This condition can occur in amyloid light-chain (AL) amyloidosis and transthyretin amyloidosis. Cardiac involvement in amyloidosis is associated with poor prognosis and can lead to various symptoms, including chest pain, HF, arrhythmias, stroke, and signs and symptoms of autonomic dysfunction. The disease also affects other organs, with symptoms such as carpal tunnel syndrome, easy bruising, macroglossia, neuropathy, and hepatomegaly. The ECG in amyloid cardiomyopathy often shows low-voltage QRS complexes. Echocardiography typically reveals bi-ventricular hypertrophy, valve thickening, bi-atrial dilatation, and diastolic dysfunction. Strain and strain rate imaging using speckle tracking can help to differentiate amyloid cardiomyopathy from sarcomeric HCM. In most cases, the pattern of LVH in cardiac amyloid is concentric and non-obstructive, but some cases can present with asymmetric obstructive forms, mimicking sarcomeric forms of HCM. CMR has a central role in the non-invasive diagnosis of cardiac amyloidosis due to its ability to provide tissue characterisation in addition to high-resolution morphologic and functional assessment. While multiple LGE patterns have been described in cardiac amyloidosis, subendocardial and transmural distributions predominate. Both patterns are present to different extents in AL and ATTR cardiac amyloidosis, with subendocardial LGE being more prevalent in AL and transmural LGE more prevalent in ATTR cardiac amyloidosis. LGE initially appears predominant in the basal segments, but as the disease advances, biventricular transmural involvement occurs [13,14]. Additionally, it’s worth noting that wild-type ATTR features almost universal involvement of cardiac structures, while hereditary ATTR involvement varies. Both types of amyloidosis can mimic HCM. On the other hand, AL amyloidosis, the most prevalent form, affects the heart in approximately 50% of cases. It often presents as a non-ischemic dilated phenotype, resulting in global impairment of cardiac function.
- **Mitochondrial cardiomyopathies:** this is a heterogeneous group of conditions that arise due to genetic mutations in the mitochondrial DNA inherited maternally. This leads to impaired energy production and affects various systems in the body, including the central nervous system, heart, and skeletal system. The symptoms of the disease can manifest at any age, from infancy to adulthood. In around 25% of patients, non-obstructive cardiomyopathy with mild concentric hypertrophy is observed, significantly worsening the prognosis. Approximately 50% of these patients develop HF, and the mortality rate reaches 70% before the age of 30. In cases of HF, cardiac transplantation may be considered a treatment option.
- **RAS-HCM:** HCM phenotype can be associated with malformation syndromes, including Noonan syndrome and LEOPARD syndrome. These syndromes belong to a group of developmental disorders called RASopathies caused by mutations in genes involved in the RAS/mitogen-activated protein kinase (RAS/MAPK) pathway. Noonan syndrome is inherited in an autosomal dominant pattern and is characterised by various congenital heart defects, including hypertrophic cardiomyopathy, which can affect up to 25% of patients. Additionally, Noonan syndrome patients may have pulmonary and aortic valve stenosis and atrioventricular septal defects. LEOPARD syndrome is an allelic variant of Noonan syndrome and is characterised by a combination of clinical features, including lentigines, electrocardiographic abnormalities, ocular hypertelorism, pulmonic stenosis, abnormal male genitalia, retardation of growth, and deafness. A recent multi-omics study in myectomy tissue from HCM patients shows activation of the RAS-MAPK signalling, suggesting that RAS-HCM and sarcomeric HCM may, in fact, share some final common pathways. In a multicentre retrospective study aimed to understand the arrhythmic progression of RAS-HCM better and pinpoint shortcomings in its management, RAS-HCM was associated with heightened morbidity and a comparable risk of SCD to sarcomeric HCM [15]. Notably, there was a discernibly low frequency of ICD implantation among RAS-HCM patients, resulting in potentially preventable sudden deaths. Prospective studies are needed to identify risk factors for SCD and develop specific recommendations for ICD implantation in RAS-HCM [16].
- **Hypertensive heart disease:** LVH caused by hypertension can be challenging to differentiate from HCM (Table 2) caused by sarcomeric mutations, as there is a frequent overlap (up to 25%) in the patterns of hypertrophy seen in both conditions. Similarly, features of HCM, such as the systolic anterior motion (SAM) of the mitral valve anterior leaflets, chordal slack, friction or impact lesions (from chronic mitral–septal contact on septum) or dynamic LVOTO due to basal septal hypertrophy can also be observed in patients with LVH caused by hypertension, especially if untreated hypertension is severe and low preload. However, SAM in hypertensive LVH occurs at the end of systole, unlike in HCM, where it occurs earlier. Several echocardiographic techniques have been used to differentiate between the two conditions [17]. Tissue Doppler imaging can show more impairment of diastolic function in HCM and lower early diastolic velocities. Two-dimensional (2D) strain echocardiography can also aid in the diagnosis, as radial strain in the mid and apical short-axis segments is commonly reduced in HCM with sarcomeric mutations. Similarly, the systolic longitudinal strain has been found to be reduced in HCM, and it has value in distinguishing between HCM and hypertensive LVH. CMR imaging can identify typical patterns of fibrosis associated with HCM. Serum markers such as norepinephrine, atrial natriuretic peptide, and brain natriuretic peptide tend to be higher in HCM patients than in those with hypertensive LVH. A thorough clinical assessment of relatives may be crucial in making a diagnosis, as the identification of HCM in family members dramatically increases the likelihood that LVH has a genetic basis.

- **Danon disease:** this is a rare X-linked dominant genetic disorder caused by a deficiency in lysosome-associated membrane protein-2 (LAMP2), leading to lysosomal storage. The prevalence of the disease may be underestimated due to difficulties in diagnosis, but LAMP2 mutations have been identified in 1–8% of patients with suspected HCM who underwent genetic testing. The disease manifests in males with severe symptoms at an earlier age, while females may develop later onset and milder symptoms due to X-linked inheritance. Diagnosis is confirmed by molecular genetic screening that reveals a LAMP2 gene mutation. Clinical suspicion of the condition should prompt testing of serum creatine kinase and liver enzyme levels, which are usually raised in this condition. ECG may show ventricular pre-excitation with Wolff–Parkinson–White (WPW)-pattern in up to two-thirds of men and less than a third of women, along with very large voltage complexes in male teenagers, raising suspicion of the condition. Echocardiography typically shows severe concentric LVH, but asymmetric septal hypertrophy has also been observed. Skeletal muscle biopsy may show intra-sarcoplasmic periodic acid-Schiff-positive vacuoles. In late stages, the disease may progress to a dilated cardiomyopathy phenotype [18,19].
- **Pompe disease:** this is a genetic disorder that follows an autosomal recessive pattern of inheritance, resulting from the deficiency of acid maltase (acid alpha [α]-glucosidase) enzyme. The condition is characterised by the deposition of glycogen in multiple organs and can present in three different forms: infantile, juvenile, and adult. The infantile form is the most severe and can lead to death within two years due to extreme LVH, HF, hypotonia, macroglossia, and hepatomegaly. While dilated cardiomyopathy can occur in some patients, the infantile form of the disease often presents with asymmetric LVH and LVOTO. In contrast, later onset forms have milder cardiac involvement and present with proximal myopathy. Diagnosis can be confirmed by demonstrating enzyme deficiency in fibroblasts, lymphocytes, and/or urine, and skeletal muscle biopsy showing vacuolar glycogen deposition. ECG can show features of LVH as well as short PR interval with pre-excitation or conduction block [20].
- **PRKAG2 cardiomyopathy:** this is a genetic disorder with autosomal dominant inheritance caused by mutations in the PRKAG2 gene, which codes for the regulatory gamma-subunit of AMP-activated protein kinase (AMPK). This condition typically affects adolescents and young adults and is characterised by muscle weakness and imaging showing LVH with global hypokinesia. While the LVH in PRKAG2 cardiomyopathy is often associated with excess glycogen deposition and can be variable in severity, it can also be asymmetric, resembling the pattern seen in HCM caused by mutations in sarcomeric genes. However, PRKAG2 cardiomyopathy is different from sarcomeric HCM in that it progresses early to systolic dysfunction and dilated cardiomyopathy. This condition may also be associated with WPW syndrome and degeneration of the conduction system [21,22]. There are no known precision-based therapies for *PRKAG2* cardiomyopathy.
- **Cori–Forbes cardiomyopathy:** this is a genetic disorder caused by mutations in the glycogen debranching enzyme (amylo-alpha-1, 6-glucosidase [AGL]) gene. It is inherited in an autosomal recessive pattern and can present in infants, adolescents, or young adults. Common clinical features of Forbes disease include muscle weakness, poor growth, and hypoglycemia. In addition, patients may develop concentric LVH, which can progress to dilated cardiomyopathy in later years [23].
- **Athlete’s heart:** in response to chronic high-intensity physical activity, the cardiovascular system activates a series of adaptative physiological mechanisms defined as the athlete’s heart, including a constellation of changes with increased biventricular mass, volume, and wall thickness. A stepwise approach to the cardiovascular assessment of athletes is essential to make sense of overlapping clinical phenotypes and eventually provide a correct differential diagnosis between HCM and adaptative cardiac response to exercise. Twelve-lead ECG enhances the sensitivity of the screening process by allowing early detection of cardiovascular conditions distinctively manifesting with ECG abnormalities. Echocardiography has a pivotal role in differentiating physiologic and pathologic responses to exercise, namely athlete’s heart, from HCM. Combining different methods, such as 2D and 3D measurements of cardiac size, volumes, wall thickness, mass index, tissue velocity, and myocardial strain imaging, cardiac ultrasound allows comprehensive morphologic and functional evaluation of the heart and distinction between physiologic and pathologic remodelling. In the presence of abnormal, uncertain, and/or controversial findings from the upstream diagnostic work up, CMR imaging can help distinguish between exercise-induced cardiac remodelling and cardiovascular pathology. CMR represents the current gold standard in the non-invasive assessment of cardiac morphology and quantification of volumes and flow and offers the opportunity for advanced myocardial tissue characterisation with excellent accuracy and precision [24,25].

## 2. Clinical Diagnosis and Imaging Tools

Timely detection and effective management are crucial for improving the prognosis of HCM. The 2020 AHA/ACC guidelines [3] recommend a comprehensive approach, starting with a detailed physical examination and thorough medical and family histories for individuals suspected of having HCM. Consideration for clinical evaluation of HCM arises when there is a family history of HCM, the presence of symptoms or cardiac events, detection of a heart murmur during physical examination, findings from echocardiography performed for other reasons, or an abnormal 12-lead ECG. A comprehensive clinical examination for HCM necessitates gathering an extensive personal and familial cardiac history that extends across three generations [26]. Pedigree analysis is particularly valuable as it helps to tease out the genetic origin of the disease and identify at-risk family members. Important attributes to consider in the family history include SCD, unexplained HF, cardiac transplantation, pacemaker and defibrillator implants, and evidence of systemic diseases such as early-onset stroke, skeletal muscle weakness, renal dysfunction, diabetes, and deafness. Pedigree analysis can also help to identify the mode of inheritance, with most genetic forms of HCM being autosomal dominant and showing affected individuals in each generation, regardless of sex. However, the recent finding of a Carter effect suggests a possible multifactorial threshold model of inheritance with sex dimorphism for liability [26]. An in-depth physical examination incorporating specific manoeuvres like the Valsalva, squat to stand, passive leg raising, or walking is also recommended. Neurological examination should be performed for syndromic HCM and myopathies (i.e., *FHL1* with contractures). Following physical examination, initial assessment often includes an ECG, which may appear normal in some cases at the initial presentation. However, it typically reveals a variable combination of LVH, ST- and T-wave abnormalities, and pathological Q-waves. Several patterns are also recognised: (i) widespread and deep T-wave inversion typical of apical and mid cavity HCM; (ii) pre-excitation with PRKAG2 and Danon (LAMP2); (iii) voltage criteria LVH with ST changes and LV strain, seen in HCM; (iv) ST elevation preceding T-wave inversion indicative of apical aneurysm; and (v) inferior Q waves indicative of extensive scarring.

The next step is performing imaging to identify LVH when clinical findings indicate its existence. Cardiac imaging is indeed essential to confirm the diagnosis, understand the underlying pathophysiology, and evaluate the risk of SCD. However, diagnosing HCM can sometimes pose unique challenges. These include patients presenting at advanced stages of the disease, with dilated, hypokinetic and/or thinned left ventricle (LV) (burnt-out phase), athletes with HCM [27], concurrent conditions associated with LVH, or isolated basal septal hypertrophy, particularly in older hypertensive individuals [28].

Echocardiography is pivotal as the primary diagnostic and monitoring tool in HCM [29]. M-mode, 2D, and Doppler transthoracic echocardiography (TTE) are commonly employed techniques, while strain imaging and 3D can help to detect subtle changes in early phenotypes. The ACC/AHA and ESC guidelines recommend the use of TTE, to be performed at rest and during Valsalva, as the initial diagnostic approach in patients with suspected HCM. TTE is also recommended for screening family members of HCM patients. TTE enables a comprehensive assessment of LV wall thickness, as well as the identification of mitral valve abnormalities, systolic anterior motion, LVOTO, left atrial enlargement, LV diastolic and systolic function, right ventricular function, and pulmonary haemodynamics. If initial findings are inconclusive, echocardiography with intravenous contrast, transoesophageal echocardiography (TOE), or cardiovascular magnetic resonance (CMR) imaging can be recommended [30,31]. TTE can also differentiate between different phenotypes of HCM, distinguishing obstructive from non-obstructive types. Tissue Doppler imaging (TDI) is an advanced tool useful to quantify the radial and longitudinal motion of the myocardium. In HCM, TDI can identify isolated reduction of systolic velocities in patients with preserved LVEF, early diagnosis before the development of overt LV hypertrophy, and presence of intraventricular dyssynchrony [32]. Strain measures myocardial deformation in multiple directions throughout the cardiac cycle. In HCM, reduction of LV global longitudinal strain (GLS) does occur in individuals with preserved LVEF and is a marker of subclinical myocardial dysfunction with demonstrated incremental prognostic value [32,33]. However, as strain-based measures are yet to be standardised and adopted into clinical HCM guidelines, GLS should be used to help distinguish HCM from cardiac amyloidosis, and athletic remodelling [31]. Three-dimensional echocardiography can aid further in assessing LV geometry, spiral distribution of hypertrophy, patterns of papillary muscle abnormalities, and recognising septal insertion of the moderator band and apical-basal muscle bundles preventing overestimation of maximal wall thickness [34]. Three-dimensional technology is also valuable in TOE to better detail the abnormalities of the mitral valve apparatus, SAM features, and underlying causes. Stress echocardiography, which involves imaging the heart during controlled exercise using an exercise bike or treadmill, can reveal hidden or latent obstruction in symptomatic patients. This is particularly useful when baseline TTE has failed to show LVOT gradients of ≥50 mmHg even when accompanied by the previously described physiological manoeuvres.

**Figure 1:**
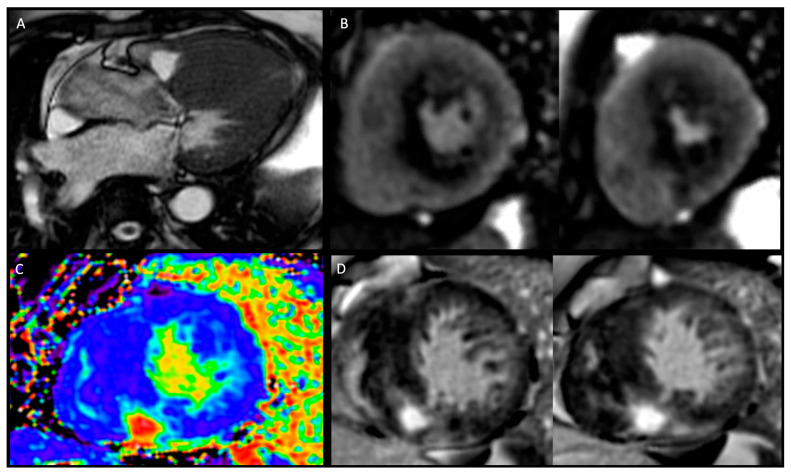
Phenotyping hypertrophic cardiomyopathy by CMR. Panel (**A**): Cine imaging—three chamber view depicting asymmetric left ventricular hypertrophy (LVH) with systolic anterior movement and left ventricular outflow tract turbulence. Panel (**B**): Adenosine stress perfusion imaging with perfusion defect in the areas of LVH and late gadolinium enhancement (LGE). Panel (**C**): extracellular volume mapping. Panel (**D**): LGE module: short-axis views showing right ventricular insertion point LGE and septal diffuse mid-wall LGE.

In the past, CMR was usually performed when echocardiography yielded inconclusive findings, but its use has become increasingly popular in recent years, and is now recommended in all patients with cardiomyopathy at initial evaluation (Class I, LoE B) [35]. While echocardiography is the first-line imaging modality and considered the standard for diagnosing HCM, it has limitations due to its dependence on adequate acoustic windows and challenges in obtaining cross-sectional images at the correct angles. Doppler assessment of LVOT mid cavity and apical gradients is crucial with echocardiography to guide management. In this regard, CMR complements echocardiography by enabling a complete and accurate assessment of all myocardial segments of the LV, here including the anterolateral wall, apical segments, septal junctions, and of the RV. It also allows evaluation of the papillary muscles (hypertrophy, abnormal insertion, feathering, and apical displacement) and detection of myocardial crypts. This comprehensive evaluation allows for precise reconstruction of cardiac size, morphology, function, and tissue characterisation. CMR with LGE is now recommended in HCM patients for diagnostic work up (Class I, LoE B) and further risk assessment (Class IIa, LoE B) [36]. Gadolinium-enhanced CMR plays a crucial role in precisely identifying myocardial fibrosis. The presence of LGE indicates replacement myocardial fibrosis and helps stratify the risk of ventricular arrhythmias and sudden cardiac death. Numerous studies have investigated the association between LGE and long-term outcomes in HCM and have consistently reported a significant positive relationship between presence of LGE and heightened risk of total and cardiovascular death, and HF. Native T1 mapping and extracellular volume fraction (ECV) are prolonged in HCM, indicating the presence of myocardial disarray and diffuse fibrosis. These values not only correlate with the risk of developing VT but also help differentiate HCM from other conditions. Tissue characterisation with CMR can also help to exclude other causes of LVH such as Fabry’s (low myocardial T1) or cardiac amyloidosis (elevated T1 and patterns of LGE). Coronary microvascular dysfunction can be further explored with stress CMR perfusion imaging as a valuable tool to detect perfusion abnormalities that might occur before the development of overt LVH or scarring in HCM gene mutation carriers due to microvascular obstruction, supply–demand mismatch, extravascular compressive forces, and elevated intraventricular pressures (Figure 1). An independent association between microvascular disease electrocardiographic abnormalities has been observed in subclinical HCM, also suggesting the arrhythmogenic potential of small vessel disease [37]. Furthermore, measurable changes in microvascular function and myocardial microstructure by diffusion tensor imaging represent novel early-phenotype biomarkers in the emerging era of disease-modifying therapy [38,39,40]. Further research is yet to be conducted to establish the role of this novel approaches [38].

Cardiac computed tomography (CCT) is not commonly used in patients with HCM. Its indications arise when there are unclear findings on echocardiography, poor acoustic windows, or contraindications for performing CMR. CCT can be performed for evaluating ischemia in patients with HCM due to its ability to assess cardiac morphology, coronary anatomy, and myocardial perfusion. However, this still needs to be improved in clinical practice as only a few centres offer dynamic perfusion CT imaging. If significant stenosis is identified in the major epicardial artery, it serves as an indication for invasive angiography. This approach facilitated the detection of underlying disease processes and enabled surgeons to develop a clear plan regarding the volume and location of the surgery, ultimately reducing the occurrence of perioperative complications. According to international guidelines [3], there is currently no compelling justification for routine invasive haemodynamic evaluation in the assessment of patients with oHCM or conducting routine coronary angiography in the general population with HCM. These invasive procedures are typically recommended when clinical and noninvasive imaging examinations do not provide sufficient diagnostic information, but obtaining such information would impact patient management. Invasive cardiac catheterisation, while not essential for diagnosis, can be employed to determine the extent of obstruction and evaluate the haemodynamics of blood flow. It is particularly recommended in cases where obstructive HCM coexists with valvular aortic stenosis. This assessment allows for the identification of diastolic abnormalities, such as increased chamber stiffness and impaired LV relaxation. Additionally, invasive angiography provides insight into the characteristics of HCM, including asymmetric hypertrophy, mitral regurgitation, and systolic anterior movement of the anterior leaflet of the mitral valve, subaortic membranes, and subaortic conus. Moreover, coronary angiography is the primary investigative tool in patients with HCM who exhibit symptoms of angina or possess risk factors for coronary atherosclerosis. It is an integral component of alcohol septal ablation and is performed before surgical myectomy. The purpose of coronary angiography in these cases is to assess the septal anatomy and identify the presence of coronary artery disease that may require intervention, beyond the scope of septal ablation.

## 3. Sudden Cardiac Death Risk Assessment and Prevention

The management of HCM requires a collaborative approach, where the patient and the healthcare team work together harmoniously [29]. This partnership entails the active involvement of the patient and relatives in decision-making processes, ensuring their understanding of the benefits, risks, action plans, and ultimate goals concerning their condition. Patient engagement has proven to enhance confidence in clinical choices and improve health outcomes based on available evidence. The primary objective of shared decision-making is to minimise the risk of new complications, slow disease progression, and improve the quality of life. Shared decision-making involves a comprehensive dialogue among the healthcare team, which includes primary care physicians, cardiologists, paediatricians, internists, nurse practitioners, and patients. Numerous studies highlight the importance of shared decision-making in HCM management, primarily due to the absence of straightforward solutions. Patient treatment options may vary depending on factors such as the need for invasive therapies to address LVOTO, genetic testing, implantation of a cardioverter defibrillator (ICD), or participation in physical activities, particularly for professional athletes. It is crucial to consider that strenuous physical exertion poses a risk of sudden cardiac death in certain individuals with HCM. However, recent evidence suggests that among individuals with HCM or those who are genotype positive/phenotype negative and are treated in experienced centres, those exercising vigorously did not experience a higher rate of death or life-threatening arrhythmias than those exercising moderately or sedentary [41]. Shared decision making holds particular significance for athletes with HCM, especially for those who aspire to resume sports activity. Many international sports organisations have strict guidelines [42] regarding athletes with HCM and other arrhythmogenic disorders. Therefore, shared decision making should not be oversimplified as a process in which athletes have sole decision-making authority. It is essential to avoid limiting shared decision making solely to patient autonomy, even in the context of athletes.

SCD, although rare (overall 1–2% rate), is the most devastating complication of HCM. Therefore, it is crucial to identify and stratify risk factors. The stratification of risk factors is primarily based on clinical symptoms, complaints, and radiological reports. Stratification in paediatric patients is different, and work up includes non-sarcomeric causes, given earlier onset, and checking for biallelic or digenic disease. In adults, both the ACC/AHA and ESC guidelines recommend secondary prevention ICD implantation for patients who meet specific criteria.

According to the AHA/ACC guidelines, established risk markers of SCD in HCM patients include prior cardiac arrest or sustained ventricular tachycardia, family history of HCM-related sudden death in first-degree relatives, unexplained syncope, maximum LV wall thickness over 30 mm, LVEF < 50%, LV apical aneurysm (with or without LGE), non-sustained ventricular tachycardia on Holter monitoring, and extensive LGE (≥15% of LV mass). Importantly, in patients aged ≥ 16 years, 5-year risk estimates of SCD can be considered to fully inform patients during shared decision-making discussions. Notably, the evolution of SCD risk assessment, including the addition of new risk markers, has resulted in the removal of abnormal blood pressure response to exercise as a routine part of the SCD risk evaluation.

According to the 2014 ESC guidelines [43] for the diagnosis and management of HCM and the 2023 ESC guidelines for the management of cardiomyopathies [35], the HCM Risk-SCD tool (HCM Risk-Kids for children and adolescents < 16 years) is recommended to assess the 5-year risk of SCD in HCM patients. The ESC risk stratification scheme shares some factors with the ACC/AHA approach, such as left ventricular wall thickness, family history of SCD, and syncope. However, the ESC model includes additional factors like age, LV outflow tract gradient, and left atrial diameter. Using the HCM Risk-SCD tool, patients can be classified as low risk (5-year risk < 4%), intermediate risk (5-year risk ≥ 4 to <6%), and high risk (5-year risk ≥ 6%) of SCD. For the low-risk group, ICD is usually not recommended. The intermediate-risk group may be considered for ICD, while the high-risk group should be considered for ICD placement. In practice, the presence of extensive LGE (≥15%) and LV systolic dysfunction (LVEF < 50%) can be used may be used in shared decision making with patients about prophylactic ICD implantation in low to intermediate risk categories. Furthermore, while the 2022 ESC Guidelines for the management of patients with ventricular arrhythmias included genetic testing to identify single or multiple sarcomeric pathogenic variants to evaluate the need for ICD implantation in intermediate risk patients [36], the 2023 ESC guidelines for the management of cardiomyopathies [35] now recommend against routine use of the presence of sarcomeric variant(s) to guide decisions around ICD implantation for primary prevention.

## 4. Therapeutic Approaches

Clinicians usually focus their preventive measures on patients presenting with overt signs and symptoms of disease, particularly in younger patients. Nevertheless, the complications of the disease have a higher chance of affecting individuals who are between the ages of 50 to 70 years old. HCM patients experience a ‘honeymoon period’, which is the time between the initial detection and the onset of severe clinical signs of the disease [1]. This period can last for over 30 years. Clinicians can exploit this temporal window to actively prevent or delay the adverse progression of the disease through medication or medically necessary procedures that can change the course of the disease. Although empirical therapeutic approaches can currently manage acute complications by reducing symptoms and improving quality of life, there are no drugs or interventions available that have been found to significantly slow the progression of LV systolic or diastolic dysfunction or prevent the transition from subclinical to overt HCM. However, a recent study by Joy et al. revealed that it might be possible to prevent the transition from subclinical to overt HCM in some individuals with genetic variants. Furthermore, positive myocardial remodelling might be more amenable to reversal at the subclinical stage of disease than in overt HCM, when cardiac hypertrophy and fibrosis are challenging to reverse. Studies to explore this possibility would benefit from a comprehensive phenotypic description of subclinical HCM, with the potential for therapeutic targeting of those individuals with early phenotypic changes. An era in which cascade screening is replaced by cascade prevention, enable by novel phenotyping, is a tempting prospect [44].

Despite being a prevalent disease, HCM lacks specific drug treatment, especially in terms of selective disease-modifying drugs. HCM was erroneously regarded as a rare disease, which has hindered the completion of large, randomised trials. As a result, most of the current guidelines are based on small observational studies, case series, or expert consensus. The present recommendations are established using empirical data from non-specific drugs like disopyramide, non-dihydropyridine calcium channel blockers, or β-blockers, administered mainly to oHCM symptomatic patients. Nonetheless, such medications provide complete relief from obstruction in only a small subgroup of patients, implying that most treated individuals are left with lingering LVOT gradients and symptoms.

For patients with oHCM whose gradient remains above 50 mmHg and remain symptomatic despite optimal drug treatment, invasive septal reduction therapies are recommended [3,43]. These therapies include either surgical septal myectomy (where possible and favourable perioperative risk in high volume centres, or, requiring other surgery such as mitral valve surgery), or catheter-based alcohol septal ablation, if not suitable for myectomy and anatomy amenable to septal ablation. However, there are potential risks associated with these treatment options, and the risk of complications is lower in specialised medical centres. Unfortunately, access to these centres may be limited for many people around the world.

Recently, new compounds have been created to treat HCM by directly targeting the hypercontractility and altered energetics of the cardiac muscle. These innovative drugs work through allosteric inhibition of myosin, the primary protein of the heart muscle responsible for generating force [45]. In this review, we will thoroughly examine the use of different categories of medications in patients with HCM (Table 3), including conventional treatment, as well as new disease-modifying treatments and emerging gene modulation approaches.

### 4.1. Conventional Treatment

Conventional treatment of HCM includes pharmacological therapy, surgery, and lifestyle modifications [46]. This approach is defined as “non-selective” because it does not target the primary mechanisms of the disease, but it acts on the consequences of the hypertrophic myocardium. Although there is no evidence that conventional drugs are able to prevent disease progression [47], they aim to manage symptoms, improve cardiac function, and reduce the risk of complications. β-blockers, calcium channel blockers, and disopyramide are the most common non-selective drugs used in HCM.

#### 4.1.1. β-Blockers

In the context of HCM, β-blockers (BBs) are commonly prescribed, specifically those without vasodilating effects. Metoprolol and atenolol are among the most widely used BBs, but nadolol and bisoprolol can also be used [48]. Currently, the choice of β-blocker is more influenced by the clinical practice of the single institution rather than specific recommendations guided by randomised evidence.

LVOTO is a common cause of exertional dyspnea, angina, and or fatigue in patients with HCM. These symptoms occur due to myocardial hypercontractility combined with a strong adrenergic drive [1]. Indeed, BBs are very effective in patients with symptomatic LVOTO, and they should be titrated focusing on symptoms to the maximally tolerated dose. On the other hand, BBs could also be very helpful in limiting symptoms in noHCM and preserved ejection fraction. This cohort of patients often complains about angina and shortness of breath, associated with microvascular ischemia, diastolic dysfunction, and elevated filling pressures. BBs help to relieve symptoms through their negative chronotropic and inotropic effects [3,43].

NoHCM is usually well tolerated, and only a small portion of individuals with this condition develop HF with reduced ejection fraction (HFrEF). In these cases, BBs can be prescribed to reduce death and HF hospitalization [49], in addition to ACE inhibitors (ACEIs), angiotensin II receptor antagonists (ARBs), angiotensin receptor-neprilysin inhibitors (ARNIs), SGLT2 inhibitors (iSGLT2s), and mineralocorticoid receptor antagonists (MRAs).

According to the ESC guidelines [35,43], BBs are first-line drugs to improve symptoms in patients with resting or provoked LVOTO (Class I, LoE B), and in patients with symptomatic noHCM and EF > 50% (Class IIa, LoE C). BBs are also recommended in noHCM and EF < 50% (Class IIa, LoE C). The 2020 American guidelines [3] confirm the recommendations and level of evidence for β-blockers in both oHCM and noHCM. Special mention should be made of asymptomatic patients with noHCM and preserved EF; in this case, the benefit of β-blockers is not proven (Class 2b, LoE C-EO).

#### 4.1.2. Calcium Channel Blockers

The CCBs used in HCM are the non-dihydropyridine agents, devoid of vasodilating effects. Dihydropyridine CCBs are generally contraindicated in HCM because systemic vasodilation may increase LVOT gradients and obstructive symptoms [50]. The hypertrophic cardiomyocyte has higher than normal cytosolic calcium concentration, leading to hypercontractility, diastolic dysfunction, and arrhythmias [51]. Non-dihydropyridine CCBs act by inhibiting L-type calcium channels, exerting negative inotropic and chronotropic effects on working myocytes and sinoatrial node cells, respectively.

Diltiazem and verapamil are the most common drugs of this class [29]. Their efficacy is similar to BBs in reducing symptoms [52]. Non-dihydropyridine agents are commonly suggested when BBs are not well tolerated. Side effects of CCBs include headache, dizziness, nausea, constipation, edema, and flushing. They can rarely lead to HF, especially in patients with high resting gradients and advanced HF, or conduction disturbances. The combination of CCBs and BBs is generally not recommended due to the risk of bradycardia and hypotension [1].

The ESC guidelines [35,43] recommend verapamil or diltiazem (Class I, LoE B) in patients with oHCM who are intolerant or have contraindications to BBs, in order to improve symptoms. They are also indicated in symptomatic noHCM with preserved ejection fraction (Class IIa, LoE C). These recommendations are also endorsed by the 2020 American guidelines [3]. Like BBs, using CCBs in asymptomatic patients with noHCM is possible but requires further evidence (Class 2b, LoE C-EO).

#### 4.1.3. Disopyramide

Disopyramide is a class IA antiarrhythmic drug exerting its effects through multiple mechanisms. It primarily acts by blocking the cardiac sodium channel INaL to reduce the influx of sodium ions into cardiac cells during the action potential, while exerting a minor effect on the peak sodium current INa, responsible for the action potential upstroke. In addition to its effect on the sodium channels, disopyramide also has a secondary blocking effect on L-type calcium channels. By blocking these channels, disopyramide reduces the influx of calcium ions into the cardiac cells during the plateau phase of the action potential. This contributes to a decrease in contractility and a reduction in myocardial oxygen consumption. Disopyramide also affects the ryanodine receptors, which are calcium-release channels located on the sarcoplasmic reticulum of cardiac cells. By blocking these channels, disopyramide helps to decrease the release of calcium from the sarcoplasmic reticulum, which leads to a decrease in diastolic calcium concentration. This effect can contribute to the relaxation of the cardiac muscle during diastole [53]. Lastly, disopyramide has a mild blocking effect on the delayed potassium current (IKr). This action prolongs the repolarization phase of the action potential. Due to this effect, disopyramide was historically used in acute coronary syndrome to prevent arrhythmias. Subsequently, this drug has found application in HCM with LVOTO. Its use is justified by its strong negative inotropic effect due to the reduction of intracellular calcium and indirectly to the reduction of sodium, which leads to increased activity of the sodium-calcium exchanger. Furthermore, disopyramide reduces the haemodynamic consequences of the Venturi effect by lowering flow velocities in the LVOT, thereby reducing the pulling force on the anterior mitral leaflet. Side effects of disopyramide are primarily due to its antagonism of muscarinic receptors. They include dry mouth, constipation, and urinary hesitancy. New clinical studies suggest a different effect of disopyramide based on the degree of electrophysiological remodelling of the cell [54]. In hypertrophic cells, the duration of the action potential is pathologically prolonged due to the increase of INaL and L-type calcium current, and the reduction of repolarising potassium currents. By inhibiting the sodium current, disopyramide appears to reduce the duration of the action potential in these cells. Therefore, it may reduce the dispersion of the action potential and prevent the development of re-entrant arrhythmias [55].

The ESC guidelines suggest using disopyramide in addition to BBs (or, if this is not possible, with verapamil) to improve symptoms in patients with resting or provoked LVOTO (Class I, LoE B). It could be used also as monotherapy, although with a lower level of recommendation (Class IIb, LoE C) to improve symptoms in patients with resting or provoked LVOTO (Class IIb, LoE C), taking caution in patient with atrial fibrillation, in whom it can increase ventricular rate response. The 2020 American guidelines also recommend the use of disopyramide in symptomatic oHCM despite treatment with BBs and non-dihydropyridine calcium antagonists (Class 1, LoE B-NR).

#### 4.1.4. Cibenzoline

Cibenzoline is a Class IA antiarrhythmic drug used for the treatment of oHCM in Japan and Korea [56]. It shares some similarities with disopyramide in terms of its mechanism of action. Like disopyramide, cibenzoline blocks sodium channels (peak INa and INaL), which leads to a reduction in intracellular calcium concentration. This effect is responsible for its negative inotropic effect and its use in managing symptoms of oHCM [57]. By reducing the LV pressure gradient and limiting LV remodelling, cibenzoline helps alleviate symptoms associated with oHCM. Cibenzoline is considered safe and well tolerated [58]. It does not significantly alter heart rate, but slightly prolongs the QT interval. In comparison to disopyramide, cibenzoline has lower inhibitory activity on muscarinic receptors. This drug is currently not mentioned in the European or American guidelines.

#### 4.1.5. Late Sodium Channel Blockers

This class of drugs consists of ranolazine and eleclazine. Their mechanism of action involves the inhibition of sodium current INaL, which led to a reduction of intracellular calcium. In hypertrophic cells, the pathologically increased INaL current prolongs the action potential duration, which predisposes to the development of early after-depolarization events. Ranolazine and eleclazine exert an antiarrhythmic function because they reduce the INaL sodium current and, consequently, the development of early after-depolarization events. Moreover, they normalise diastolic calcium, improving the relaxation of myocardium in HCM and probably lowering the incidence of delayed after-depolarizations, which are diastolic calcium-release events.

Two trials have attempted to demonstrate the effect of ranolazine and cibenzoline on symptoms in HCM. In the RESTYLE HCM trial, ranolazine was tested on symptomatic patients with noHCM, but it did not show a significant improvement in functional capacity as measured by cardiopulmonary exercise testing [59]. However, an improvement in the arrhythmic profile and lower levels of BNP were observed. The LIBERTY HCM trial aimed to test eleclazine in symptomatic patients with and without LVOTO. The study was prematurely terminated following the discouraging results seen in other trials [60]. These drugs are currently not mentioned the European or American guidelines.

#### 4.1.6. Angiotensin II Receptor Antagonists

ARBs are among the most used medications for the treatment of hypertension and HF. By binding to the AT1 receptor expressed on cardiomyocytes and fibroblasts, angiotensin II promotes the development of hypertrophy and fibrosis [61]. Therefore, the use of ARBs should slow down the progression of the disease and promote reverse remodelling, particularly by improving diastolic function [62]. However, the available studies on the use of ARBs in HCM have failed to demonstrate a clear benefit in terms of reducing cardiac mass and its fibrotic component [63].

New encouraging perspectives come from the multicentre, double-blind, phase II trial VANISH. This study tested valsartan in a population of patients with early-stage HCM, characterized by limited alterations on echocardiography. Standardized changes from baseline to year 2 in LV wall thickness, mass, and volumes; left atrial volume; tissue Doppler diastolic and systolic velocities; and serum levels of high-sensitivity troponin T and N-terminal pro-B-type natriuretic protein were integrated into a single composite z-score as the primary outcome. The results published at a 2-year follow up showed a reduction in the worsening of diastolic function and in the development of hypertrophy in the group treated with valsartan compared to the placebo. These findings seem to confer a primary importance to early screening, although further evidence is desirable in this regard [64]. According to the current American and European guidelines [3,43], ARBs are recommended in patients with HCM and systolic dysfunction and progression towards end stage. However, caution is advised when considering the early usage of ARBs in cases of obstruction, as they have the potential to affect both preload and afterload, which could worsen the obstruction.

#### 4.1.7. Angiotensin Receptor Neprilysin Inhibitors

ARNIs have been shown to be among the most effective HF drugs, by improving both systolic and diastolic function. New evidence suggests that this class may induce reverse remodelling in a hypertrophic heart [65]. By inhibiting both the RAAS and the natriuretic peptides system, ARNIs block pathways involving calcineurin-NFAT, JNK, ERK1/ERK2, and p38, mediating an anti-hypertrophic signalling [62]. This effect may be less pronounced in the presence of long-standing hypertensive heart disease or ischemic heart disease, which are characterized by the presence of fibrotic tissue. ARNIs are currently used in the context of HCM with HFrEF. New horizons will be outlined by the results of the SILICOFCM study (NCT03832660), aimed at extending the use of ARNIs to symptomatic patients with noHCM and preserved ejection fraction [66].

#### 4.1.8. Septal Reduction Therapy

Septal reduction therapy (SRT) represents an important option for severely symptomatic patients with LVOTO [67]. There are two established techniques: myectomy and alcohol septal ablation. Originally, myectomy involved widening the LVOT by resecting the myocardium at the level of the anterior basal septum. Nowadays, it has evolved to extend beyond the mitral–septal contact point, including the mid-ventricular septum as well. This procedure not only reduces LVOTO gradient and Venturi effect, but also the pushing forces responsible for systolic anterior motion and, therefore, mitral insufficiency. When mitral regurgitation is caused by morphological alterations of the valve apparatus, myectomy can be combined with mitral repair (or, more rarely, replacement). In patients with a history of atrial fibrillation undergoing myectomy, it is possible to perform the Maze procedure to reduce the occurrence of arrhythmic events. Although left atrial appendage ligation is often also performed, this does not protect against thromboembolic events in HCM with AF due to underlying atrial myopathy.

Septal myectomy is considered the current gold standard due to its excellent results in terms of effectiveness and safety [29]. Most patients undergoing myectomy report an improvement in symptoms and quality of life, supported by an increase in peak VO_2_ during cardiopulmonary exercise testing. These results are correlated with the immediate abolition of LVOTO and with the reduction in ventricular filling pressures, leading to reverse left atrium remodelling and modest regression of LVH. The main complications are complete heart block requiring a permanent pacemaker, ventricular septal defect, and aortic regurgitation.

Alcohol septal ablation (ASA) involves injecting alcohol into a septal perforator artery: the area supplied by the affected vessel will undergo necrosis and fibrosis, resulting in a reduction in wall thickness. Contrast echocardiography is essential to assess if the coronary anatomy is compatible with the procedure. If the contrast is not localized exclusively in the basal septum and adjacent mitral–septal contact point, the patient is not eligible. This technique is mostly reserved for frail patients with a high surgical risk or contraindications to surgery [68]. ASA is the first alternative to myectomy [69]. Both procedures are associated with an improvement in functional status, although there are no randomized comparison trials available. ASA has the advantage of shorter hospitalization and less invasiveness [70]. The risk of mortality and complications is similar; however, ASA is burdened with a higher incidence of AV blocks, scar-related ventricular arrhythmias, and a slower and less uniform reduction in LVOTO gradient. Finally, it is not indicated in patients with surgically correctable valve abnormalities or in patients with excessively hypertrophied or thinned septum [71].

According to the European guidelines, SRT is reserved for patients with LVOTO peak gradient > 50 mmHg with recurrent exertional syncope (Class IIa, LoE C) or severely symptomatic (NYHA III-IV, Class I, LoE B) despite optimised medical therapy. Myectomy is preferred when surgical repair of other lesions is necessary (Class I, LoE C). Mitral valve repair is indicated in cases of moderate to severe regurgitation not caused by SAM (Class IIa, LoE C). The American guidelines recommend SRT in patients with symptomatic oHCM despite optimised medical therapy (Class 1, LoE B-NR). In presence of associated cardiac disease requiring surgery, myectomy is recommended (Class 1, LoE B-NR); if myectomy is contraindicated, ASA is preferred (Class 1, LoE C-LD). Furthermore, these guidelines open the possibility of early intervention (NYHA II) in case of pulmonary hypertension, atrial enlargement with episodes of atrial fibrillation, low functional capacity, or children/young adults with resting LVOTO > 100 mmHg (Class 2b, LoE B-NR). SRT can also be considered as an alternative to escalating medical therapy in symptomatic and eligible patients, after evaluating and collectively discussing the pros and cons of the choice (Class 2b, LoE C-LD). Both guidelines emphasize the importance of experienced high-volume HCM centres. A surgeon should perform at least 10 myectomies or ASA procedures per year to ensure adequate expertise and maintain a low level of risk.

#### 4.1.9. Lifestyle Interventions

Patients with HCM need to adopt certain lifestyle adjustments [72]. These include avoiding dehydration, excessive alcohol consumption, extreme temperatures (hot/cold), and heavy meals, as these situations could trigger or worsen LVOTO. For the same reasons, European guidelines also discourage using venous or arterial dilators, nitrates, and phosphodiesterase-5 inhibitors. Digoxin should also be avoided due to its positive inotropic effect. The role and methods of physical activity are highly debated. New evidence highlights the role of aerobic exercise in reverse remodelling of the ventricle. In fact, exercise reduces levels of angiotensin II, BNP, and aldosterone, mimicking the effects of many pharmacological therapies.

Current European guidelines discourage high-intensity physical activity, although they emphasize the importance of a healthy lifestyle [43]. American guidelines recommend physical activity from mild to moderate intensity, as it improves the quality of life and cardiopulmonary fitness (Class 1, LoE B-NR) [3]. They consider physical activity of any intensity reasonable (Class 2a, LoE C-LD) for individuals with a positive genotype and negative phenotype. Finally, the practice of high-intensity sports for patients with HCM can be considered reasonable (Class 2b, LoE C-LD), following a comprehensive evaluation of the risk of sudden cardiac death and a shared discussion to be repeated annually.

Some recently completed or ongoing trials may change the above recommendations in the near future [41]. The LIVE-HCM prospective cohort study compared the outcome associated with vigorous exercise, moderate exercise, or sedentary lifestyle in HCM individuals. This trial enrolled 1660 participants who were followed for three years, excluding participants with NYHA class III or IV symptoms. HCM subjects exercising vigorously did not experience a higher rate of the composite endpoint of death, resuscitated sudden cardiac arrest, arrhythmic syncope, and appropriate shock from an implantable cardioverter defibrillator than those exercising moderately or those who were sedentary [41].

**Figure 2:**
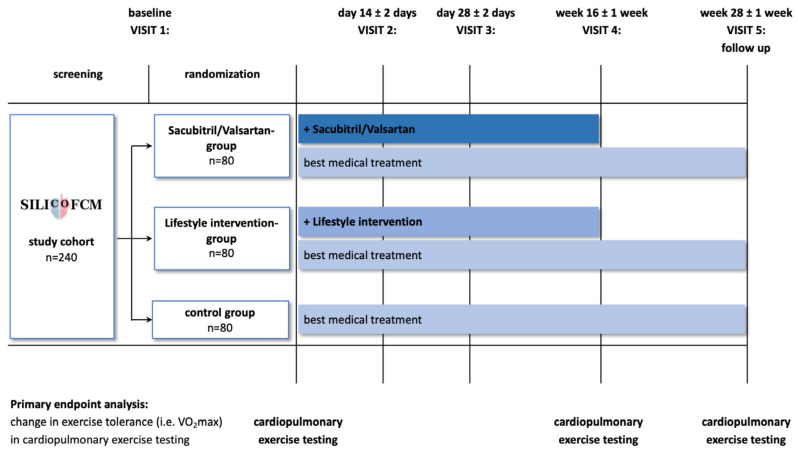
SILICOFCM trial design. Reproduced with permission from Tafelmeier M.et al., 2020 [66].

Another trial is the phase II SILICOFCM study (Figure 2). The objective of this ongoing trial is to evaluate the benefits of pharmacological therapy with ARNIs compared to lifestyle interventions in patients with noHCM. Both ARNIs therapy and lifestyle interventions with exercise training and dietary supplementation with inorganic nitrate, could have a potential role in improving symptoms, cardiac performance, and reverse remodelling, although this is still not fully clarified [66].

### 4.2. Novel Therapeutic Approaches

**Figure 3:**
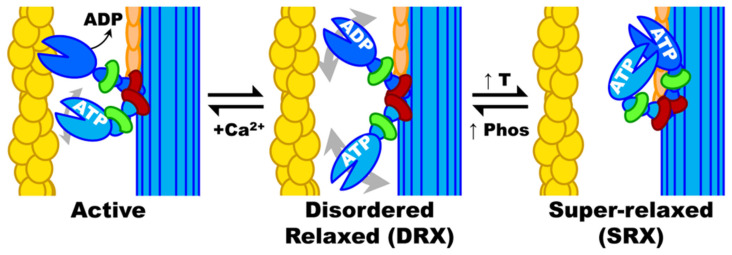
Myosin biochemical and structural states. Actin filaments are activated by Ca^2+^ (which binds to troponin, not shown) allowing myosin heads to be activated by actin binding followed by rapid turnover of ATP and molecular force generation. When Ca^2+^ is removed, and ATP replaces ADP, myosin heads return to DRX, which can then cycle to and from SRX. When myosin heads are not ordered on the thick filament backbone (as in SRX), they are disordered and can rotate about in the interfilament space, as depicted by grey double-headed arrows in DRX. Actin, yellow; myosin heavy chain, blue; essential light chain, green; regulatory light chain, red; myosin binding protein C, orange; DRX, disordered relaxed; SRX, super-relaxed. Reproduced with permission from Phung et al., 2018 [74].

The myocardium regular contractions are achieved by carefully balancing performance and energy expenditure [73]. Myosin molecule activation state plays a crucial role in both determining and influencing myocardial contraction by being able to shift between two conformations during relaxation: namely a sequestered “super relaxed state” (SRX) and a “disordered relaxed state” (DRX) [56]. The difference lies in ATPase (adenosine triphosphatase) activity: in SRX conformation, myosin has low enzyme activity. On the other hand, in DRX state, more myosin heads are available to interact with actin, with greater enzyme activity (Figure 3).

In unaffected patients, energy demand seems to be the main factor influencing the transition between the two states. Energy is in fact conserved in SRX conformation. This can be achieved by preventing the formation of unnecessary cross bridges. The DRX state instead allows a greater performance at the cost of higher energy consumption [75]. Experimental evidence indicates that a pathological shift of the myosin equilibrium towards the DRX state causes impaired relaxation in HCM. This increases the number of myosin heads available to interact with actin, which in turn leads to both an enhanced contraction and higher energy expenditure [76]. Moreover, mutations in R403Q (the first identified mutation in HCM) and various HCM variants in MYBPC3 have been linked to impaired relaxation, hyperdynamic contractions, and increased energy consumption by the sarcomeres [77]. This is therefore linked to the balance between SRX and DRX states of myosin. Interestingly these characteristics often precede the development of LVH.

#### 4.2.1. Mavacamten

Mavacamten is a novel specific myosin inhibitor that has the ability to restore the equilibrium between SRX and DRX conformations of myosin, reducing the myocardial force of contraction and consumption of ATP by myosin [78]. Experimental studies on mouse cardiac myofibrils have shown that mavacamten has a dose-dependent reduction in ATPase activity. This highlights its direct action on myosin, targeting its enzymatic activity. Mavacamten demonstrated an inhibitory effect on the basal rate of ADP release in bovine cardiac myosin, reducing it by 50% without affecting the rate of ADP release when myosin was in an actin-associated state. The number of myosin heads available for interaction with actin during the transition from the weakly to the strongly bound conformation is greatly reduced. Mavacamten effectively prevents these myosin heads from taking part in the actomyosin chemo-mechanical cycle. By decreasing ATPase activity, mavacamten leads to a reduction in the power generated by the sarcomere [79]. Furthermore, after 20–26 weeks of treatment, histological analysis of the myocardium highlighted a decrease of 80% of fibrosis. This raises mavacamten to a disease-modifying drug status, as fibrosis is a characteristic feature of the disease. However, this effect is not seen after the onset of hypertrophy [80]. As follows, Mavacamten maximum effect is exerted at the early stages of disease progression.

Experimental studies on Mavacamten administration (10 μM) to Yucatan minipigs carrying R403Q mutation showed a significant restored the percentage of SRX, shifting SRX/DRX ratio back to wild-type animals. Mavacamten stabilizes myosin heads in the closed SRX states and reduces the available myosin molecules for interaction with actin, preventing unnecessary actomyosin interaction, preserving myocardial physiological performance. Mavacamten can therefore restore the normal expression of genes involved in contractility and metabolism in HCM, restoring the physiological transcription pathways. The improvement in functional parameters resulting from mavacamten treatment correlates with the normalization of the SRX/DRX balance. This suggests that hyperdynamic contractility seen in HCM is likely promoted by an increased proportion of myosin in the DRX conformation, while delayed relaxation is mediated by a reduction of myosin heads in the SRX state [81].

#### 4.2.2. Mavacamten Trials

Mavacamten improves performance in patients with oHCM by reducing LVOTO. Several studies have been conducted in this area, with some still ongoing. One notable study is the PIONEER-HCM trial, which was a phase II, multicentre, open-label trial involving 21 patients with oHCM [82]. The participants were divided into two cohorts (A and B) and each participant underwent a 12-week treatment cycle with once-daily oral mavacamten, followed by a 4-week post-treatment phase. In cohort A, the patients were treated with mavacamten to gain insights into the pharmacokinetic and pharmacodynamic relationship of the compound. The starting dose was based on the patient’s weight, with a dose of 10 mg/day for patients weighing 60 kg or less and 15 mg/day for those weighing more than 60 kg. On the other hand, cohort B received lower concentrations of mavacamten, with a dose of 2 mg/day for all patients. The dosage variation aimed to evaluate whether different doses could influence the expected effect [83]. The study results indicate that 12 weeks of mavacamten treatment had beneficial effects on patients with oHCM, by significantly reducing the degree of post-exercise LVOTO. This correlated with improvement in exertional capacity and symptom relief. The effect on LVOTO reduction was more prominent in cohort A, with a mean reduction of 82% compared to 29% in cohort B. Higher doses of mavacamten may therefore yield better outcomes for patients. Mavacamten administration was not exempt from adverse effects although most of them were reported as mild (80%). Most commonly, it caused a reduction in LVEF below 50% and atrial fibrillation [83]. The PIO-NEER-OLE study (NCT03496168), an ongoing 5-year extension of the PIONEER-HCM trial, is currently being conducted. This prospective, open-label, multicentre trial aims to assess the long-term effects of mavacamten. Interim analysis conducted after the first year indicates sustained efficacy of mavacamten throughout 156 weeks of follow up [84]. The PIOONER-OLE study is anticipated to conclude in November 2023 [85].

MAVERICK-HCM (Mavacamten in adults with symptomatic non-obstructive hypertrophic cardiomyopathy) was a phase II, multicentre, double-blind, randomized, placebo-controlled study that enrolled 59 patients with symptomatic noHCM, preserved left ventricular ejection fraction (LVEF ≥ 55%), increased NT-proBNP (≥300 pg/mL), and exertional symptoms classified as New York Heart Association (NYHA) functional class II/III. The patients were randomly assigned to three different cohorts [86]. Cohort 1 consisted of 19 patients who received a pharmacokinetic-adjusted dose of mavacamten, with a target serum drug concentration of approximately 200 ng/mL. Patients in Cohort 2 were instead treated with higher doses of mavacamten to achieve serum drug concentrations averaging around 500 ng/mL. Finally, Cohort 3 received a placebo. The treatment duration for all cohorts was 16 weeks. Results showed that NT-proBNP decreased by 53% in the mavacamten group compared to 1% in the placebo group. Additionally, cardiac Troponin-I decreased by 34% in the mavacamten group, whereas there was a 4% increase in the placebo group. Further analyses demonstrated that Mavacamten improved echo parameters of diastolic function [86].

EXPLORER-HCM was a phase III, multicentre, randomized, double-blind, placebo-controlled trial conducted in 68 clinical cardiovascular centres across 13 countries [87]. The study aimed to evaluate the efficacy and safety profile of mavacamten, which proved to be superior to placebo in both primary and secondary endpoints. For the primary endpoint, 45 out of 123 patients (37%) in the mavacamten group experienced a 1.5 mL/kg per min or greater increase in peak oxygen consumption (pVO_2_) with at least one-point reduction in NYHA class or an increase of over 30 mL/kg per min in pVO_2_ with no changes in NYHA class. In contrast, only 22 out of 128 patients (17%) in the placebo group achieved the primary endpoint. Additionally, patients receiving mavacamten showed improvements in post-exercise LVOT gradient, pVO_2_, and patient-reported health status. Significantly, almost 30% of patients (32 out of 117) treated with mavacamten achieved a complete pharmacological response, defined as a reduction in LVOT gradient to less than 30 mmHg with marked improvement in symptoms. This response was observed in less than 1% of patients (one out of 126) in the placebo group. Notably, the greatest benefit was observed in patients who were not receiving BBs. Treatment with mavacamten also resulted in consistent improvements in patients’ reported health status, with greater symptom relief and improved quality of life compared to the placebo group. There was a strong correlation between the magnitude of improvement in health status and the extent of improvement in pVO_2_, emphasizing the association between mavacamten treatment and enhancements in patient well-being and quality of life. Improvement in health status was reversed 8 weeks after the completion of treatment. A reduction in LVOT was associated with a significant decrease in LVEF. However, the normalization of LVEF after the conclusion of therapy was not immediate, suggesting that, although rare, the improvement in the health status of oHCM patients could be counteracted by the slow reversal of the drug’s effect due to its long half-life.

VALOR-HCM was a randomized controlled trial conducted by Desai et al. [88], which investigated whether the addition of mavacamten to maximally tolerated background medical therapy could reduce guideline eligibility for SRT in highly symptomatic oHCM patients. The trial included 112 highly symptomatic patients with oHCM who met the guideline criteria for SRT. These patients had a mean age of 60 ± 12 years, with 51% being male, and 93% classified as NYHA functional class III. The study was conducted between July 2020 and October 2021 at 19 sites in the United States. After 16 weeks of treatment, the results showed that 77% of the patients randomized to placebo (43 out of 56) continued to meet the guideline criteria for SRT or chose to undergo the procedure. Only 7.9% of the mavacamten-treated patients (10 out of 56) met the guideline criteria or elected to undergo SRT (treatment difference of 58.9% [95% CI: 44.0–73.9%]; *p* < 0.001). The addition of mavacamten significantly reduced the eligibility for SRT in these highly symptomatic oHCM patients. Furthermore, this study demonstrated that mavacamten treatment resulted in a significant reduction in LVOT gradients. As in other trials, there were improvements observed in NYHA functional classification and quality-of-life measures among the mavacamten-treated patients. VALOR-HCM was not able to provide long-term safety outcomes due to its focus on a 16-week treatment period. To assess long-term safety, efficacy, and clinically guided dosing, the ongoing 5-year extension study, MAVA-LTE (Mavacamten Long Term Extension; NCT03723655), is currently underway. An interim analysis has already been performed for this study. During the 36-week follow up evaluated, mavacamten has been shown to achieve significant reduction in both resting and Valsalva-induced LVOTO gradients, with changes reported as −27.4 ± 33 mmHg and −45.7 ± 39.9 mmHg, respectively [89].

ODISSEY-HCM (NCT05582395) is an ongoing randomized, double-blind, phase III study to evaluate mavacamten in adults with symptomatic noHCM compared to placebo. The composite primary endpoints include change from baseline in Kansas City Cardiomyopathy Questionnaire (23 item) Clinical Summary Score and change from baseline in peak oxygen consumption (pVO_2_) at week 48. The recruitment phase is ongoing [90].

Overall, trial results for mavacamten have showcased positive safety and efficacy profile. As a result of these findings, the Food and Drug Administration, on April 2022, and the European Commission, on June 2023, have approved this drug for the treatment of symptomatic (NYHA, class II-III) oHCM in adult patients. Furthermore, on 15 June 2023, FDA approved a revised label for mavacamten to reflect drug ability to reduce the need or eligibility for SRT in patients with obstructive HCM as evidenced by the VALOR-HCM trial. Importantly, since mavacamten was initially approved, its prescription details have carried a “Boxed Warning” about the potential risk of HF. This warning stems from data indicating that its use might lead to a reduction in LVEF, which could potentially result in HF due to systolic dysfunction. Of note, mavacamten, as primarily metabolized by CYP2C19, exhibits a wide-ranging elimination half-life dependent on the CYP2C19 phenotype, spanning from 72 to 533 h. For poor metabolisers or patients awaiting genotyping results, the recommended starting dose is 2.5 mg. The dose can only be increased beyond 5 mg once genotyping has confirmed that the patient is not a poor metabolizer [91]. For all other phenotypes, the starting dose is 5 mg. Dose adjustments throughout the treatment, initially at 4-week intervals and subsequently at 12-week intervals, are directed by LVOT gradient and LVEF, as detailed in decision trees in the summary of product characteristics.

New ESC guidelines [35] delivered class IIa recommendation for mavacamten in addition to a beta blocker (or, if this is not possible, with verapamil or diltiazem), or as monotherapy, to improve symptoms in adult patients with resting or provoked LVOTO.

#### 4.2.3. Aficamten

A second myosin inhibitor/allosteric modulator currently under development for the treatment of HCM is CK-274, also known as Aficamten [92]. While Aficamten shares a similar molecular effect with mavacamten, it binds to a different regulatory site on the myosin heads. Aficamten possesses certain pharmacokinetic advantages over mavacamten. It exhibits a shorter half-life of approximately 2 days, allowing for a faster washout in the presence of side effects. Additionally, aficamten does not interact with the enzymes CYP2C19 and CYP3A4, reducing the likelihood of drug–drug interactions. These characteristics contribute to the potential clinical benefits of aficamten in the management of HCM [1].

#### 4.2.4. Aficamten Trials

Phase II REDWOOD-HCM study evaluated the efficacy of aficamten in oHCM patients.

Patients with oHCM and LVOT gradients ≥ 30 mmHg at rest or ≥50 mmHg with Valsalva were randomized 2:1 to receive aficamten (*n* = 28) or placebo (*n* = 13) in two dose-finding cohorts. Most patients treated with aficamten (78.6% in Cohort 1 and 92.9% in Cohort 2) achieved the treatment target of reducing the resting gradient below 30 mmHg and the post-Valsalva gradient below 50 mmHg by week 10. The mean differences were −40 ± 27 mmHg and −43 ± 37 mmHg in Cohorts 1 and 2, respectively, at rest (*p* = 0.0003 and *p* = 0.0004 versus placebo, respectively). During Valsalva, mean differences were −36 ± 27 mmHg and −53 ± 44 mmHg in the two cohorts, respectively (*p* = 0.001 and *p* < 0.0001 versus placebo, respectively). The incidence of adverse events was similar between the treatment arms, with a negligible reduction in LVEF observed in patients who were administered aficamten. LVEF was transiently reduced below 50% in only one treated patient [93]. FOREST-HCM (previously known as REDWOOD-OLE; NCT04848506) is the 5-year extension study of REDWOOD-HCM [94]. At 48 weeks, aficamten was shown to be safe and well tolerated in patients with oHCM. This trial highlighted a substantial reduction in peak resting and Valsalva LVOT-gradients from baseline to week 48 (resting mean ± SD: –32 ± 28 mmHg; Valsalva mean ± SD: –47 ± 28 mmHg). There was a modest reduction in LVEF from baseline to week 48 (–5 ± 3%) and no patients experienced an aficamten-related reduction in LV ejection fraction < 50%. There was a substantial improvement in functional class, as by week 48, 88% of patients experienced ≥1 NYHA functional class improvement while none had functional worsening.

**Figure 4:**
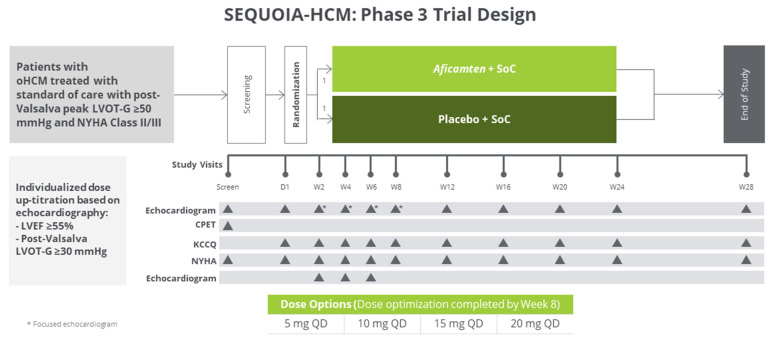
SEQUOIA-HCM trial design. Reproduced with permission from Incorporated, C. 2021 [97].

These data support the continued development of aficamten, which is currently being investigated in the large randomized, placebo-controlled, phase III clinical trial SEQUOIA-HCM (NCT05186818). This study aims to recruit a total of 270 symptomatic oHCM patients. The primary endpoint is the change in pVO_2_ measured by cardiopulmonary exercise testing from baseline to week 24 (Figure 4). Following the positive results from Cohort 3 of REDWOOD-HCM, patients whose background therapy includes disopyramide, are eligible for enrolment [95]. The recruitment phase is ongoing [96].

MAPLE HCM (NCT04349072) is phase 3 multicentre, randomized, double blind active-comparator clinical trial of aficamten compared to metoprolol in patients with symptomatic oHCM. A summary of completed trials is reported in Table 4.

## 5. Personalized Therapy

The management of HCM represents a major challenge due to the substantial genotypical variability observed in this disease. To better assess the efficacy of pharmacological therapies, human-based computational methodologies have emerged as powerful tools in recent years. By integrating experimental and clinical data, simulation studies have shed light on HCM pathophysiology, phenotypic expression, arrhythmic risk, and response to pharmacological interventions. These advancements have enhanced our mechanistic understanding of the disease and paved the way for personalized therapy in HCM [98].

Passini et al. utilized human experimental data on HCM to develop and calibrate an electrophysiological model of HCM cardiomyocytes [99]. This model successfully elucidated key mechanisms contributing to arrhythmia development in HCM and identified potential pharmacological targets. Reactivation of the overexpressed ICaL (L-type calcium current) was identified as a crucial factor driving repolarization abnormalities in HCM. Through simulated human drug trials, the researchers investigated anti-arrhythmic strategies tailored to the HCM phenotype. Selective ICaL block demonstrated high efficacy in suppressing pro-arrhythmic abnormalities but compromised calcium transient amplitude. Conversely, multichannel blockage of sodium–calcium exchanger, late sodium current INaL, and ICaL exhibited a more favourable efficacy profile without negative effects on systolic calcium.

In the majority of HCM cases, the disease is caused by genetic mutations affecting sarcomeric proteins. In silico studies have explored the primary effects of point mutations on sarcomere contractility and their propensity for arrhythmogenesis [100]. Computational techniques ranging from molecular dynamics to spatially explicit sarcomere modelling have been employed. By focusing on early human pathophysiology associated with HCM mutations, independent of compensatory responses and long-term remodelling, effective targets can be identified. Modulating these targets pharmacologically could aid in resolving the disease phenotype. Leveraging information on the impact of myosin mutations on cellular contractility, Margara et al. employed an in silico model of human electromechanical cardiomyocytes to simulate the effects of the novel myosin inhibitor mavacamten and investigate its mutation-specific efficacy in HCM [101].

Germline editing is a promising strategy for addressing monogenic diseases by preventing the transmission of mutations to future generations. The development of genome editing technologies, such as CRISPR/Cas9, has generated considerable enthusiasm for their therapeutic potential in cardiovascular diseases. However, several challenges are still ahead before a clinical implementation can be realized [102].

Current techniques, including CRISPR/Cas9, induce double-stranded DNA breaks at specific genetic loci, which triggers the cell’s intrinsic repair mechanisms. Recent advancements have demonstrated the feasibility of high-fidelity gene repair in human embryos carrying HCM mutations. In a study, oocytes from healthy women were inseminated with sperm from a male patient carrying a heterozygous MYBPC3 mutation. By simultaneously injecting a mutation-specific CRISPR/Cas9 system during early metaphase, the mutation was successfully edited in 100% of cases. Importantly, this technique also eliminated mosaicism, a phenomenon where sister cells within the same embryo possess different genotypes [103]. However, off-target effects remain a significant concern.

Another promising gene-based therapeutic approach for monogenic diseases is allele-specific gene silencing. This technique typically involves the transduction of an adenovirus vector containing short-interfering ribonucleic acid (siRNA) segments designed to suppress the expression of a specific pathogenic allele. This method, known as ribonucleic acid interference (RNAi), has shown efficacy in pre-clinical models for attenuating the phenotype of mutations associated with conditions such as catecholaminergic polymorphic ventricular tachycardia, HCM, and restrictive cardiomyopathy [104]. However, this approach is better suited for conditions caused by gain-of-function mutations (e.g., MYH7) and may not apply to the entire spectrum of HCM (e.g., MYBPC3).

Reichard et al. [105] explored the efficacy of two distinct genetic therapies in mice carrying the heterozygous HCM pathogenic variant myosin R403Q. Their approach focused on both a genetic therapy approach involving an adenine base editor (ABE8e, allowing for precise modification of the genomic DNA sequence) and delivery of RNA-guided Cas9 nuclease using AAV9. In the first case, a single dose of this dual-AAV9 system successfully corrected the pathogenic variant in over 70% of ventricular cardiomyocytes, leading to the restoration of normal cardiac structure and function. Furthermore, this correction remained durable over time. The second genetic therapy using RNA-guided Cas9 nuclease aimed to inactivate the pathogenic allele entirely. Although effective, it exhibited dose-dependent toxicities. This study highlighted the substantial potential of single-dose genetic therapies to correct or silence pathogenic variants, thus preventing the development of HCM. In fact, they achieved a long-lasting in vivo base editing of the pathogenic single-nucleotide variant in myosin, which is highly and selectively expressed in cardiomyocytes. Moving forward, the further development and translation of this approach hold significant promise for correcting the most severe human pathogenic variants.

## 6. Artificial Intelligence: Hype or Hope?

In a study conducted by Tison et al. [106] use of artificial intelligence-enhanced electrocardiogram (AI-ECG) readings was investigated for assessing disease status and treatment response in patients with oHCM. The two algorithms used were independently created by UCSF and Mayo Clinic [107]. The study was based on data from the PIONEER-OLE trial and revealed a significant correlation between AI-ECG HCM scores and disease status, as measured by reductions in LVOT gradients and NT-proBNP levels over time in patients receiving mavacamten. The observed longitudinal associations between the AI-ECG HCM score and disease parameters likely reflect changes in the raw ECG waveform that can be detected by AI-ECG and are indicative of the pathophysiology and severity of HCM. This study presents a novel paradigm in which AI-ECG, which can be conveniently implemented remotely using smartphone-enabled electrodes, may enable the assessment of disease status and treatment response [108,109]. Future investigations can further evaluate the utility of this approach in guiding drug titration to improve patient safety and outcomes.

## 7. Conclusions

Just a few years ago, treatment for HCM was restricted to symptomatic management. The use of non-selective medications like β blockers and calcium channel blockers, alongside septal reduction surgery, ICDs for primary and secondary prevention of SCD, and lifestyle modifications, constituted the mainstays of HCM treatment for a considerable period. However, advancements in our understanding of the pathophysiological processes and molecular foundations of the disease have led to the development of specific new drug classes. These have the potential to impact not just symptoms, but also the progression of the disease itself. Emblematic of this shift in paradigm are myosin modulators, such as mavacamten and aficamten. In addition, significant breakthroughs may arise from the fields of genetics and biotechnology, heralding a transformation as captivating as it was previously unthinkable. One such example is the use of genome-editing technologies like CRISPR/Cas9, which can potentially repair the genome of embryos, exploiting the monogenic nature of HCM. Other possibilities encompass the modulation of gene expression by siRNAs that inhibit the expression of the disease-causing allele. In the future, artificial intelligence could also play a significant role in this field, contributing to minimally invasive and remote monitoring tools that could enhance risk stratification schemes and facilitate early therapeutic interventions. In summary, we are witnessing a true revolution in the therapeutic approach to HCM. HCM patients will soon benefit from an entirely new range of more specific and efficacious drugs. The dawn of precision medicine has indeed truly arrived.

## Figures and Tables

**Table 1:** Red flags for differential diagnosis between sarcomeric HCM and phenocopies.

	Inheritance Pattern	Ancillary Signs and Symptoms	ECG Abnormalities	Laboratory Findings	Echocardiography	CMR Imaging
**Sarcomeric HCM**	AD	Uncommon	- High QRS voltages - LV strain pattern - Giant TWI - Q waves of pseudo-necrosis		- Moderate-to-severe LVH (asymmetrical and septal but potentially found at any location) - 10–20% can have RVH - Diastolic dysfunction - LVOT obstruction - Mitral valve abnormalities (mitral SAM, leaflets elongation, dysplasia, prolapse, chordal elongation, laxity, and hypermobility) - Papillary muscle abnormalities (hypertrophy, splaying and apical displaced insertion) - Atrial enlargement - Apical aneurysm	- LGE at RV insertion points and intramural patchy more frequently observe at sites of hypertrophied segments - Perfusion defects - Increased values of T1 mapping indices - Increased T2 mapping/T2w hyperintensity in hot-phase of disease
**Anderson–Fabry** **disease**	X-linked	- Visual impairment - Sensorineural deafness - Paranesthesia/sensory abnormalities - Angiokeratoma - Higher stroke risk - Males > females, but females can be affected (lyonization) from mild conduction disease to overt oHCM	- Short PR interval - Pre-excitation - AV block	- In males, low or undetectable alpha-1-galactosidase - Proteinuria with/without reduced glomerular filtration rate	- Concentric LVH ((but can have LVOTO) - Increased atrioventricular valve thickness - Increased RV free wall thickness - Global hypokinesia (with/without LV dilatation)	- Basal-mid infero-lateral LGE - Low T1 mapping values (accumulation/hypertrophy phases) - T1 pseudonormalisation (advanced stage) - Progressive T1 mapping dispersion
**Cardiac** **amyloidosis**	AD (for h-TTR)	- Carpal tunnel syndrome (bilateral) - Visual impairment - Paranesthesia/sensory abnormalities -Autonomic dysfunction	- Low QRS voltage - AV block -Pseudoinfarct pattern - Bundle branch block	Proteinuria with/without reduced glomerular filtration rate	- Ground-glass appearance of myocardium - Thickened interatrial septum, atrioventricular valve thickness, RV free wall - Pericardial effusion - Global hypokinesia (with/without LV dilatation) - relative longitudinal apical sparing (cherry apex)	- Diffuse subendocardial LGE (*zebra* pattern) - Abnormal T1 nulling - Increased T1 mapping indices - Increased T2 mapping values (AL)
**Mitochondrial cardiomyopathies**	X-linked or matrilinear inheritance	- Sensorineural deafness - Learning difficulties/mental retardation - Visual impairment - Muscle weakness	- Short PR interval - Preexcitation - Abnormal CPET with low VO_2_ max	- ↑ CK - ↑ AST, ALT - Lactate	Global hypokinesia (with/without LV dilatation)	- Large amount non-ischemic LGE pattern, mostly confined to the basal LV inferolateral wall
**Hypertensive heart disease**	None	Uncommon	Isolated LVH	Microalbuminuria	- LVH symmetrical or eccentric (mild to moderate). - Normal systolic and diastolic function	Non-specific findings
**Danon** **disease**	X-linked	- Learning difficulties/cognitive impairment - Visual impairment	- Short PR - Pre-excitation - AV block - Extreme LVH (Sokolow > 100)	- ↑ CK - ↑ AST, ALT	- Extreme concentric LVH - Global hypokinesia (with/without LV dilatation)	- Large amount subendocardial or transmural scarring, with typical septal sparing
**Athlete’s heart**	None	Uncommon	Isolated LVH TWI in anterior precordial leads with J point elevation (consider ethnicity)		- LVH (mild to moderate) - Normal systolic function - Normal diastolic function	- Absence of LGE or junctional pattern - Low/normal T1 mapping indices

AD, autosomal dominant; ALT, alanine aminotransferase; AST, aspartate aminotransferase; AV, atrioventricular; CK, creatine kinase; CPET, cardiopulmonary exercise testing; ECG, electrocardiogram; HCM, hypertrophic cardiomyopathy; LGE, late gadolinium enhancement; LV, left ventricle; LVH, left ventricular hypertrophy; LVOTO, left ventricular outflow tract obstruction; RV, right ventricle; RVH, right ventricular hypertrophy; TWI, T-wave inversion.

**Table 2:** Criteria to differentiate HCM from hypertensive heart disease.

Imaging Features (Echo, CMR, CT)	HCM	Hypertensive Heart Disease
**LVH**	Severe, asymmetric IVS/ILW ratio > 1.3	Mild (<15 mm, except blacks and chronic renal failure), concentric or midly asymmetric
**LVOTO**	Frequent	Rare
**Sigmoid septum**	Rare	Frequent
**Mitral valve and papillary muscle abnormalities**	Frequent	Rare
**Crypts**	Common	Rare
**Basal-apical muscle bundles**	Frequent	Rare
**Severe longitudinal systolic dysfunction**	Frequent	Rare
**Severe strain abnormalities**	Frequent	Less frequent
**LGE**	Frequent, RV insertion points, and intramural with patchy enhancement being the most common pattern	Less frequent, non-subendocardial, no specific pattern

CMR, cardiovascular magnetic resonance; HCM, hypertrophic cardiomyopathy; ILW, infero-lateral wall; IVS, interventricular septum; LGE, late gadolinium enhancement; LVH, left ventricular hypertrophy; LVOTO, left ventricular outflow obstruction.

**Table 3:** Main drugs used or tested in HCM.

Class of Drug and Mechanism of Action	Drug and Daily Dose	Side Effect	Patients	Notes
BBs Blockade of β-receptors	Metoprolol 25–200 mg Atenolol 25–100 mg Bisoprolol 1.25–10 mg Nadolol 20–80 mg	Hypotension Bradycardia Bronchoconstriction Fatigue Limb ischemia	oHCM, noHCM	To titrate focusing on symptoms to the maximally tolerated dose
CCBs Blockade of L-type calcium channels	Verapamil 120–240 mg Diltiazem 120–240 mg	Headache, dizziness Constipation Flushing HF Conduction disturbances	oHCM, noHCM	Combination of CCBs and BBs is generally not recommended
Disopyramide (Class IA antiarrhythmic) Blockade of INaL. Other minor effects on peak INa, L-type calcium channels, ryanodine receptors and IKr	300–600 mg	Antagonism of muscarinic receptors (dry mouth, constipation, urinary hesitancy, etc.)	Refractory symptomatic oHCM despite BBs or CCBs	It can increase ventricular rate response in patients with atrial fibrillation (use concomitantly with AV blocking agent)
Cibenzoline (Class IA antiarrhythmic) Blockade of INaL and peak INa	100–400 mg	Lower inhibitory activity on muscarinic receptors than disopyramide	oHCM	Used in Japan and Korea. Not listed in ESC or AHA/ACC Guidelines
Late sodium channel blockers Blockade of INaL	Ranolazine Eleclazine	Dizziness Headache Nausea	Not approved	Not listed in ESC or AHA/ACC Guidelines
Potassium channel blockers Dofetilide Sotalol		Caution renal failure, reduce dose Monitor QTc interval as risk of life-threatening QT prolongation and TdP		Particularly useful for AF Sotalol in low doses has beta blocking effects and higher doses class III Singh–Vaughan Williams effects Exhibits reverse use-dependent effects (i.e., more potent when bradycardic)
ARBs Blockade of AT1 receptor	Valsartan 80–320 mg	Hypotension Cough Hyperkalaemia Worsen kidney function	noHCM with HFrEF	Further evidence is desirable in early HCM (VANISH)
ARNIs Blockade of AT1 receptor and neprilysin	Sacubitril/Valsartan 24/26 to 97/103 mg	Hypotension Cough Hyperkalaemia Worsen kidney function	noHCM with HFrEF	Further evidence is desirable in symptomatic noHCM with HFpEF (SILICOFCM study)
Mavacamten (myosin inhibitor) Allosteric inhibition of cardiac myosin ATPase	2.5–15 mg	Decrease in ejection fraction	NYHA II-III oHCM (in America)	Inducer of CYP3A4, CYP2C9, and CYP2C19. Long half-life (7–10 days)
Aficamten (myosin inhibitor) Allosteric inhibition of cardiac Myosin ATPase	Not established	Decrease in ejection fraction	Not yet approved	Absence of interaction with CYP3A4, CYP2C9, and CYP2C19. Short half-life (2 days)

ARB, angiotensin receptor blocker; ARNI, angiotensin receptor neprilysin inhibitor; AT1, angiotensin type 1; ATP, adenosine triphosphate; BBs, beta-blockers; CCBs, calcium channel blockers; HCM, hypertrophic cardiomyopathy; HFpEF, heart failure with preserved ejection fraction; HFrEF, heart failure with reduced ejection fraction; noHCM, nonobstructive HCM; oHCM, obstructive HCM, TdP, torsade de pointes.

**Table 4:** Completed studies on Mavacamten and Aficamten treatment in HCM patients.

Trial	Study Design	Study Population	Dose and Timeline	Sample Size	Results
**EXPLORER-HCM** [76]	Randomized double blind placebo control	Obstructive HCM NYHA II–III	Mavacamten 2.5–15 mg 30 weeks	251	Primary endpoint achieved in 37% of mavacamten patients vs. 17% placebo patients
**MAVERICK-HCM** [75]	Randomized double blind placebo control	Non-obstructive HCM NYHA class II–III	Mavacamten 16 weeks	59	Median NT-proBNP reduced by 53% in mavacamten group vs. 1% in placebo group Median TnI reduced by 34% in mavacamten group vs. 4% in placebo group
**REDWOOD-HCM** [81] **Cohorts 1 and 2**	Randomized double blind placebo control	Obstructive HCM NYHA II–III	Aficamten 5–15 mg (Cohort 1) 10–30 mg (Cohort 2) 10 weeks	41	Resting LVOTO gradient reduction: median difference of −40 ± 27 mmHg −43 ± 37 mmHg Post-Valsalva LVOTO gradient reduction: −36 ± 27 mmHg −53 ± 44 mmHg
**REDWOOD-HCM** **Cohort 3** [84]	Open label	Obstructive HCM NYHA I–III On disopyramide	Aficamten 5–15 mg 10 weeks	13	Resting LVOTO gradient reduction: −28 ± 3.2 mmHg Post-Valsalva LVOTO gradient reduction: −27 ± 5.9 mmHg
**REDWOOD-HCM** **Cohort 4** [83]	Open label	Non-obstructive HCM NYHA II–III	Aficamten 5–15 mg 10 weeks	41	NT-proBNP reduction (mean reduction by 66%, *p* < 0.0001), TnI reduction (−21%, *p* < 0.01)
**VALOR-HCM** [77]	Randomized double blind placebo control	Obstructive HCM, referred or under active consideration for SRT	Mavacamten 2.5–15 mg 32 weeks	112	After 16 weeks, 17.9% of mavacamten patients eligible to SRT vs. 76.8% in placebo group (*p* < 0.001)
**PIONEER-HCM** [71] **Cohort A**	Open label	Diagnosed with HCM, resting LVOT gradient ≥ 30 mmHg and post-exercise peak LVOTO gradient ≥ 50 mmHg	Mavacamten 10–15 mg 12 weeks	11	pVO_2_ improvement: +3.5 mL/kg/min, (95%CI:1.2;5.9); Peak exercise LVOTO gradient reduction: −90 mmHg (95%CI: −138; −41)
**PIONEER-HCM** [71] **Cohort B**	Open label	Obstructive HCM NYHA II–III	Mavacamten 2–5 mg/die 12 weeks	10	pVO_2_ improvement +1.7 mL/kg/min, (95%CI: 0.0–3.3) Peak exercise LVOTO gradient reduction: −25 mmHg (95%CI: −47; −3.0)
**FOREST-HCM** [82]	Open label	Obstructive HCM	Aficamten 5–20 mg 48 weeks	38	Resting LVOTO gradient reduction: −32 ± 28 Resting LVOTO gradient reduction: −47 ± 28
**MAVA-LTE** [78]	Open label	Obstructive HCM	Mavacamten 2.5–15 mg 36 weeks	137	Resting LVOTO gradient reduction: −27.4 ± 33 Resting LVOTO gradient reduction: −45.7 ± 39.9

HCM, hypertrophic cardiomyopathy; NYHA, New York Heart Association; LVOTO, left ventricular outflow obstruction.

## Data Availability

Not applicable.
